# Dissolved Carbon Dioxide: The Lifespan of *Staphylococcus aureus* and *Enterococcus faecalis* in Bottled Carbonated Mineral Water

**DOI:** 10.3390/biology12030432

**Published:** 2023-03-10

**Authors:** Michael Schalli, Sabine Platzer, Rainer Schmutz, Petra Ofner-Kopeinig, Franz F. Reinthaler, Doris Haas

**Affiliations:** 1 Department for Water-Hygiene and Micro-Ecology, D&R Institute of Hygiene, Microbiology and Environmental Medicine, Medical University of Graz, 8010 Graz, Austria; 2 Institute for Medical Informatics, Statistics and Documentation, Medical University of Graz, 8010 Graz, Austria; 3 Applied Hygiene and Aerobiology, D&R Institute of Hygiene, Microbiology and Environmental Medicine, Medical University of Graz, 8010 Graz, Austria

**Keywords:** *Staphylococcus aureus*, *Enterococcus faecalis*, bottled water, carbon dioxide, carbonation, capping process

## Abstract

**Simple Summary:**

The consumption of bottled mineral water has been rising in the last decades. During the water bottling process, the risk of bacterial contamination with pathogen bacteria is a well-known problem. In this study, the influence of different carbon dioxide concentrations, in mineral water bottles, on the growth of *Staphylococcus aureus* and *Enterococcus faecalis* was investigated. Inside an Austrian mineral water bottling factory, a sum of 160 glass bottles with different carbon dioxide concentrations (3.0 g/L, 5.5 g/L, and 7.0 g/L) were capped after artificial contamination, stored at room temperature (20 °C), kept away from light, and dried in the laboratory. Water samples from freshly opened bottles were filtrated and cultivated on agar media to determine the number of colony-forming units over a period of 30 days. After the 23rd day, no more cultivable bacteria were detected in the samples from the freshly opened bottles. This observation leads to the conclusion that carbonated mineral water is an inappropriate habitat for *Staphylococcus aureus* and *Enterococcus faecalis.*

**Abstract:**

During the process of mineral water production, many possible contamination settings can influence the quality of bottled water. Microbial contamination can originate from different sources, for example, the ambient air, the bottles, the caps, and from the bottling machine itself. The aim of this study was to investigate the influence of three different carbon dioxide (CO_2_) concentrations (3.0 g/L, 5.5 g/L, and 7.0 g/L; 20 bottles each) in bottled mineral water on the bacterial growth of *Staphylococcus aureus* (*S. aureus*) and *Enterococcus faecalis* (*Ent. faecalis*). The examined mineral water was artificially contaminated before capping the bottles inside the factory. After a specific number of days, water samples were taken from freshly opened bottles and after filtration (100 mL), filters were placed on Columbia Agar with 5% Sheep blood to cultivate *S. aureus* and Slanetz and Bartley Agar to cultivate *Ent. faecalis*. The respective colony-forming units (CFU) were counted after incubation times ranging from 24 to 120 h. Colony-forming units of *S. aureus* were not detectable after the 16th and 27th day, whereas *Ent. faecalis* was not cultivable after the 5th and 13th day when stored inside the bottles. The investigation of the bottles that were stored open for a certain amount of time with CO_2_ bubbling out showed only single colonies for *S. aureus* after the 5th day and no CFUs for *Ent. faecalis* after the 17th day. A reduction in the two investigated bacterial strains during storage in carbonated mineral water bottles means that a proper standardized disinfection and cleaning procedure, according to valid hygiene standards of industrial bottling machines, cannot be replaced by carbonation.

## 1. Introduction

The Codex Alimentarius Austriacus [1] and the Austrian regulations for mineral and well water [2] describe the requirements for the production of bottled mineral water in its carbonated and non-carbonated forms. *Escherichia coli* (*E. coli*), coliform bacteria, *Pseudomonas aeruginosa* (*P. aeruginosa*), and *Enterococcus* sp. must not be detectable in 250 mL of a bottled water sample, while all cultivable bacteria within the bottles must not originate from the production process [1]. The absence of coliform bacteria and fecal coliforms indicates the good quality of the investigated water, whereas determining the presence of *Staphylococcus aureus* (*S. aureus*) is not directed. A dependence of bacterial growth on pH value present in different matrices was shown in recent studies, with *S. aureus* having a more sensitive parameter to pH value than *Staphylococcus epidermidis* [3]. In 2005, a study described a dependency on the carbon dioxide (CO_2_) concentration with the growth rate of *S. aureus* at an incubation temperature of 37 °C. *Staphylococcus aureus* were cultivated on blood agar and exposed to pure CO_2_, a standard anaerobic gas, or air, over a period of 24 h [4]. Recent studies on methicillin-resistant *S. aureus* (MRSA) were concerned with antibacterial material and pathogeny [5,6,7,8,9,10,11,12]. The survival of *Escherichia coli*, *P. aeruginosa*, *Enterobacter cloacae*, and *Klebsiella pneumonia* were investigated in bottled still water and demonstrated a reduction of colony-forming units (CFUs) over a period of twenty days [13]. An investigation of *P. aeruginosa* and *Ent. faecalis* biofilms on carvacrol was recently described by Mechmechani et al. The combination of pepsin and trypsin with carvacrol treatment was more efficient than treatment with carvacrol alone [14]. Healthcare-associated infections caused by *Ent. faecalis* and *Enterococcus faecium* with resistance to multiple antimicrobial compounds can be challenging [15]. The World Health Organization (WHO) recently proclaimed that “we are entering a post-antibiotic era” owing to the intense use of antibiotics over the previous decades for agricultural and therapeutic purposes [16].

In 2020, Mohamed et al. showed that different bottled water brands in Nairobi, Kenya were contaminated with multidrug-resistant bacteria [17]. To determine the presence of microorganisms in water, different methods like multitube fermentation, filtration with subsequent plate cultivation, metagenomics analysis, biobased sensor solutions, and flow cytometry have previously been developed [18,19,20,21,22,23]. An often applied method to detect waterborne bacteria is the heterotrophic plate count (HPC) [24,25,26]. In 2012, Duranceau et al. investigated the influence of storage conditions for bottled water using the HPC. No significant increase in HPCs was found with bottles stored inside the house at room temperature or in a fridge (2 °C). The bottles stored on the porch or in a car trunk showed increased microbial growth [27]. Venieri et al. investigated the microbial contamination of non-carbonated bottled water in Greece. *Enterococcus spp*. was present in four out of the ten examined brands [28]. Plastic containers tended to be more permeable to external oxygen, which led to higher numbers of bacteria inside plastic bottles than in glass fabricates [13]. The findings of a recently published study show that no enterococci were present in bottled water sold in Mureş County in Romania. Maximum contamination of 4000 CFU/mL was found in flat spring bottled water [29]. The presence of *S. aureus* in approximately 25% of all investigated samples of drinking water in São Paulo was reported by Santos et al. in 2020. All water samples met the bacteriological water quality criteria according to Brazilian regulations, although 27% of the *S. aureus*-positive samples carried the mecA gene, indicating potential methicillin resistance [30]. Isolates of *S. aureus* in rural drinking water specimen was investigated in 1980 and showed no correlation with the presence of total coliform bacteria. However, 40% of the examined samples were positive for *S. aureus* [31]. In Nigeria, multidrug-resistant strains of *S. aureus* were isolated from clinical and drinking water sources [32]. The irrigation of *S. aureus*-infected chronic wounds with tap water has been a topic of discussion in the past [33]. Nagoba et al. suggested using boiled water, distilled water, or packed mineral water instead of tap water because of the risk of adding pathogenic microorganisms to the infected wound [34]. Hospital-associated urinary tract infections caused by MRSA are potentially life-threatening for catheterized individuals. For this reason, *S. aureus* should be avoided in the hospital environment [35]. An investigation of *S. aureus* growth in tryptic soy broth containing acidic hot spring water (60%, pH 3.9) was realized by Akiyama et al. CFUs were ten times lower than in tryptic soy broth alone after incubation for 24 h [36]. Slightly acidic electrolyzed water was found to be a growth-inhibiting habitat for *S. aureus* and other pathogenic bacteria [37]. The biofilm formation of *S. aureus* related to food and food packing materials has been intensely investigated in the past. The influence of pH value, temperature, and surface kind are the predominant factors for *S. aureus* growth [38,39,40,41]. A study, which investigated the mycological and bacteriological quality of bottled water in Ethiopia, showed that about 40% of all bottled water samples were unsuitable for human consumption. The carbonated drinking water samples were reported as free of *S. aureus*, thermotolerant coliforms, *Escherichia coli*, *Salmonella*, and *Shigella* with no indication given for CO_2_ concentration [42]. Around 14% of all water samples derived from bottled water brands in Ile-Ife Nigeria were contaminated with *S. aureus*. The authors concluded that bottled water provided by small companies was of unacceptable bacteriological quality [43].

In the literature, different potential mechanisms of CO_2_ affecting microorganisms are described [44,45,46,47]. A decrease in intracellular pH caused by a decrease in extracellular pH during CO_2_ exposure, when pH homeostatic systems are exceeded, can cause cell damage [48,49]. Garcia-Gonzales et al. described the effects of low pH and the possible influence of CO_2_ on the integrity of the cell membrane in 2010 [50,51,52]. The destruction of the cell membrane can cause intracellular material leakage [53], while high-concentration CO_2_ exposures decrease the activity of some key enzymes, which impacts cell metabolism [54,55]. A study investigating the effect of high concentrations of CO_2_ on key genes and protein expression was performed by Zhao et al. in 2016 [56]. Carbon dioxide caused reactive nitrogen species accumulation and resulted in inhibiting the transport of intracellular electrons in denitrifying microorganisms [57]. The aim of this study was to determine the effect of three different concentrations of CO_2_ (3.0 g/L, 5.5 g/L, and 7.0 g/L), which are quite common concentrations for mineral water with reduced carbonation, medium sparkling water, and sparkling water in Austria, on the bacterial growth of *S. aureus* and *Ent. faecalis* over a storage period of 30 days. *S. aureus* and *Ent. faecalis* were chosen as they are contaminants during the filling and capping process in bottled mineral water.

## 2. Materials and Methods

### 2.1. Preparation of Reference Strains for Inoculation

The reference strains used in this study were obtained from commercially available sources: *Ent. faecalis* ATCC 6057 and *S. aureus* ATCC 25923 (American Type Culture Collection (ATCC), Manassas, VA, USA), and were shipped in glass vials.

The reference strains were inoculated with a sterile loop on Tryptic Soy Agar (TSA) (VWR**^®^** International GmbH, Vienna, Austria) and incubated at 37 °C for 24 h. Subsequently, one colony of each bacterial strain was transferred into sterile Tryptic Soy Broth (VWR**^®^** International GmbH, Vienna, Austria) and incubated for 24 h at 37 °C. For further application, the suspended bacterial medium was centrifuged (3000× *g*) twice with the addition of sterile NaCl (0.5%) solution (5 mL) each, after removing the liquid parts of the samples between the centrifugation steps and at the end of the procedure. The bacterial count was determined using a DensiCheck Plus (bioMerieux**^®^** Austria GmbH, Vienna, Austria) and found to be 10^9^ colony-forming units (CFUs). A dilution series using NaCl (0.5%) solution was performed to reduce the bacterial count from 10^9^ CFUs to 10^3^ CFUs.

### 2.2. Preparation of Artificial Contaminated Water Samples

For the examined mineral water samples, the values for pH and conductivity were measured according to the respective standards (ÖNORM EN ISO 10523:2012 [58] and ÖNORM EN 27888:1993 [59], respectively) with a 712 Conductometer (Methrom**^®^** Inula GmbH, Vienna, Austria), and a Memotitrator (Metrohm**^®^** Inula GmbH, Vienna, Austria).

During the routine filling process of 250 mL glass bottles inside a mineral water bottling plant in Austria, three different concentrations of carbon dioxide containing mineral water were added (40 bottles with 3.0 g/L CO_2_, 80 bottles with 5.5 g/L CO_2_, and 40 bottles with 7.0 g/L CO_2_), shown in Table 1. Before capping the bottles, 80 bottles of mineral water were artificially contaminated with 1 mL of *Ent. faecalis* suspension and 80 bottles with 1 mL of *S. aureus* suspension. The bacterial concentrations of the inoculations were ~40 CFU/100 mL of *Ent. faecalis* for 3 g/L, 5.5 g/L, and 7.0 g/L bottles and for the open bottle; ~30 CFU/100 mL of *S. aureus* for 3.0 g/L and 240 CFU/100 mL for 7.0 g/L bottles, ~40 CFU/100 mL for the 5.5 g/L bottles and ~20 CFU/100 mL for the open bottles. The capped bottles were immediately transported to the laboratory and stored for further investigation at room temperature (20 °C). The open stored bottles were protected with a loose alumina cap for CO**_2_** release and stored at 20 °C inside a shaded box. Differences in starting concentrations for each bacterial strain were caused by varying days of inoculation and preparation of the suspensions.

### 2.3. Cultivation of Target Bacteria

**Figure 1:**
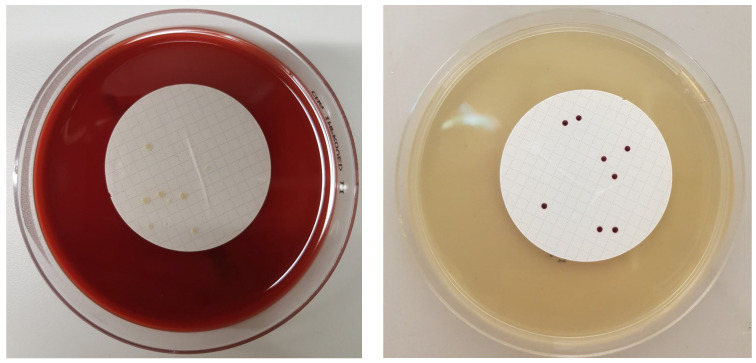
*Staphylococcus aureus* colonies on Columbia Agar with 5% Sheep blood (**left**) and *Ent. Faecalis* colonies on Slanetz and Bartley Agar (**right**).

For the cultivation of the target bacteria, a new bottle was opened on each respective day (shown in Table S1 in the Supplementary Materials). After opening the artificially contaminated bottle, water samples (100 mL) were filtrated through a sterile mixed cellulose ester filter (47 mm diameter and 0.45 µm pore size; EZ-Pak, Merck Chemicals and Life Science GmbH, Vienna, Austria, EZHAWG474) under vacuum, and the filter was subsequently placed on Columbia Agar with 5% Sheep blood (BD**^®^** Austria GmbH, Vienna, Austria) for *S. aureus* contaminated samples and on Slanetz and Bartley Agar (VWR^®^ International GmbH, Vienna, Austria) for *Ent. faecalis*, shown in Figure 1, and incubated for 120 h at 37 °C (every filtration and cultivation step was performed in duplicate). The measurement uncertainty of *Ent. faecalis* and *S. aureus* was determined according to DIN EN ISO 19036:2019 (*S. aureus*: 31.3% and *Ent. faecalis*: 15.2%) [60]. The CFU counting was performed five times (after 24 h, 48 h, 72 h, 96 h, and 120 h). To validate the respected bacterial colonies, Gram staining and the latex agglutination test were performed for selected colonies. The Thermo Scientific™ Staphaurex™ Plus Latex Agglutination Test (Thermo Fisher Scientific, Munich, Germany) was used to validate the *S. aureus* colonies, according to the manufacturer’s instructions. Using the Thermo Scientific™Streptex™ Latex Agglutination Tests (Thermo Fisher Scientific, Munich, Germany), selected *Ent. Faecalis* colonies were validated according to the manufacturer’s instructions. To avoid errors during the sample preparation, a method blank was performed for every experiment with autoclaved distilled water.

### 2.4. Gram Staining

Gram staining was performed using an IUL PolyStainer (10005300/768) and Gram-colors from Sigma-Aldrich**^®^**, Darmstadt, Germany (Gram’s crystal violet solution 1.09218.2500; Lugol’s solution 1.00567.2500 and Gram’s safranine solution 1.09217.2500). Microscopy was realized using a Carl Zeiss AG, Oberkochen, Germany, Axio-imager A1, according to the manufacturer’s instructions.

### 2.5. Data Analysis

Statistical analysis was performed, and charts were generated using SPSS Statistics 28.0 (IBM, Armonk, NY, USA). To show the relationship between the decrease in samples and time, a Kaplan–Meier analysis was performed. To assess the differences between the samples with different CO_2_ concentrations, the log-rank tests were calculated. For *Ent. faecalis* and *S. aureus*, we considered a cut-off value of CFU < 2.

## 3. Results

Analysis of the pH and electrical conductivity showed that the non-carbonated mineral water, obtained from the filling company in a glass bottle, had an electrical conductivity of 2024 ± 68 µS/cm and a pH value of 6.4 ± 0.2. The addition of CO_2_ to the mineral water changed the pH to 5.9 ± 0.1 for 3.0 g/L CO_2_, pH 5.6 ± 0.1 for 5.5 g/L CO_2_, and pH 5.5 ± 0.1 for 7.0 g/L CO_2_, with no changes for electrical conductivity.

**Figure 2:**
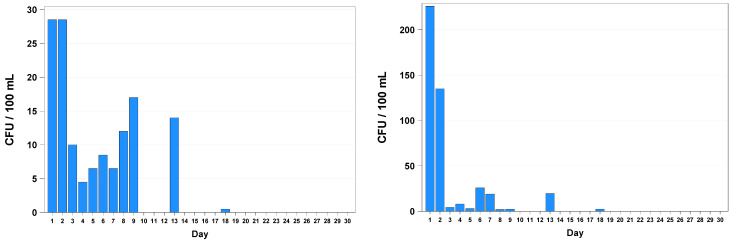
Mean values of colony-forming units (*S. aureus*) over a period of 30 days for closed bottles (**left**: 3.0 g/L CO_2_; **right**: 7.0 g/L CO_2_).

The overall incubation time was 120 h for *S. aureus* and *Ent. faecalis*. Colony-forming units were counted after 24 h, 48 h, 72 h, 96 h, and 120 h, which can be seen in Table S1 in the Supplementary Materials. The results for *S. aureus* colony counts for 3.0 g/L and 7.0 g/L CO_2_ concentrations are shown in Figure 2. The bottles with 7.0 g/L CO_2_ were inoculated with a higher load of *S. aureus*. A steep decrease in CFUs was obtained within three days of storage at this CO_2_ concentration. The number of CFUs counted from the 3.0 g/L CO_2_ bottles, over approximately 18 days, shows a count between ~2 CFUs and ~30 CFUs.

**Figure 3:**
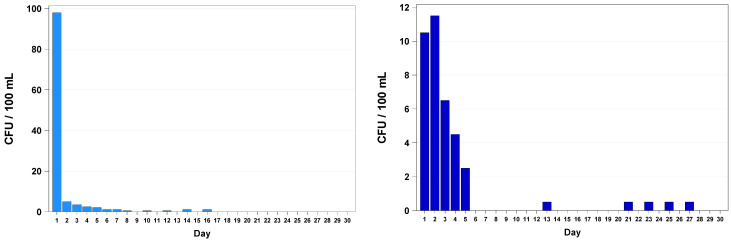
Mean values of colony forming units (*S. aureus*) over a period of 30 days for closed and open bottles (**left**: 5.5 g/L CO_2_ closed bottle; **right**: 5.5 g/L CO_2_ open bottle).

The count of CFUs for 5.5 g/L CO_2_-containing bottles (open and closed storage) is shown in Figure 3. Bottles opened after five hours of storage (5.5 g/L CO_2_) showed a mean count of 98 CFUs, which is more than double the inoculated bacteria and might be an outlier in this case. After the second day of storage, counts for the CFU drop to a mean of five. The CFU counts derived from open stored bottles were around 11 CFUs on the first two days, which dropped to zero after the 6th day when detecting single colonies for *S. aureus* on the 13th, 21st, 23rd, 25th, and 27th day. Single CFUs of *S. aureus* were also detected in samples derived from closed stored bottles (5.5 g/L CO_2_) on the 8th, 10th, 12th, 14th, and 16th days.

**Figure 4:**
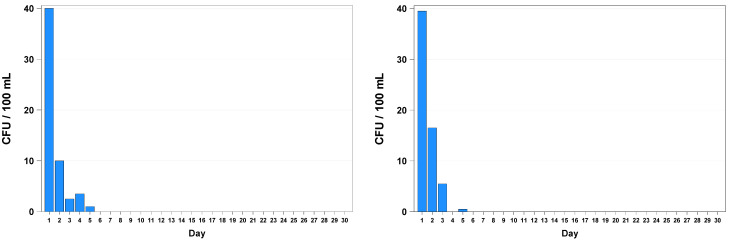
Mean values of colony units (*Ent. faecalis*) over a period of 30 days for closed bottles (**left**: 3.0 g/L CO_2_; **right**: 7.0 g/L CO_2_).

The CFU counts for *Ent. faecalis* at CO_2_ concentrations of 3.0 g/L and 7.0 g/L are shown in Figure 4. A decrease in CFUs was observed within six days for non-cultivable bacteria (*Ent. faecalis*). After the fourth day of storage, only one CFU was observed for the sample derived from 7.0 g/L CO_2_-containing bottles. The difference in the reduction of CFUs within five days of storage at these two concentrations of CO_2_ seems to be marginal.

**Figure 5:**
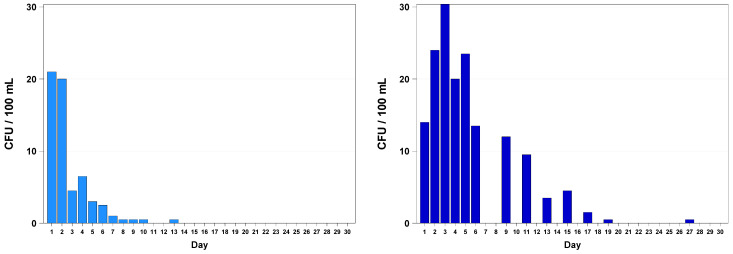
Mean values of colony forming units (*Ent. faecalis*) over a period of 30 days for closed and open bottles (**left**: 5.5 g/L CO_2_ closed bottle; **right**: 5.5 g/L CO_2_ open bottle).

In Figure 5, the CFU counts derived from open and closed stored bottles with a concentration of 5.5 g/L CO_2_ are shown. The samples from the closed bottles show a decrease in CFUs until the 8th day and drop to zero until the 11th day. The open stored bottles showed an increase of CFUs during the first three days with a slight decrease until the 19th day of storage. A single CFU was observed on day 27.

**Figure 6:**
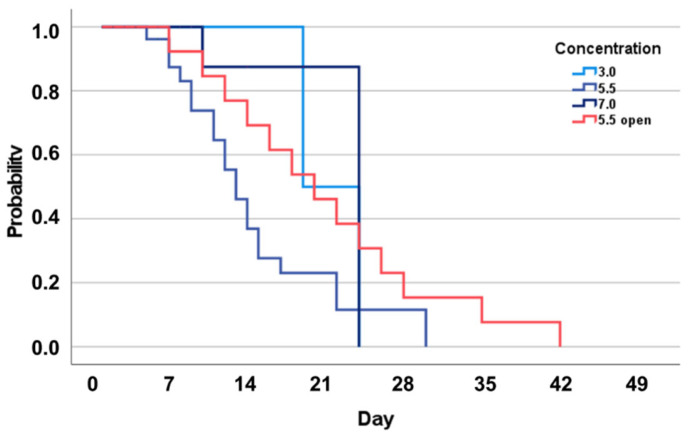
Time to the event (CFU falls below 2) for different CO_2_ concentrations of CFUs from *S. aureus* for closed and open stored bottles.

Statistical analysis was performed to determine the significance between the observed CFU counts at the different concentrations of CO_2_ (Figure 6). In the case of *S. aureus* values between 3.0 g/L CO_2_ and 5.5 g/L CO_2_ and between 5.5 g/L CO_2_ and 7.0 g/L CO_2_ were found to be significant (*p* < 0.001), according to the Kruskal–Wallis test. The median level of CFUs decreased from 10 CFU/100mL (3.0 g/L CO_2_) to 7 CFU/100mL (7.0 g/L CO_2_), whereas the median level for 5.5 g/L CO_2_ was lower with 1 CFU/100mL.

**Figure 7:**
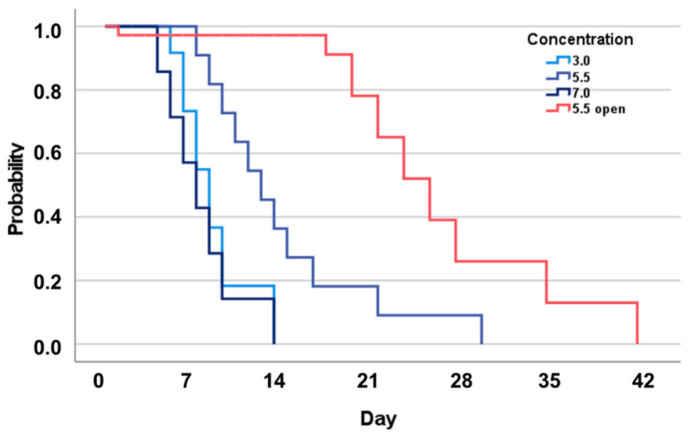
Time to event (CFU falls below 2) for different CO_2_ concentrations of CFUs from *Ent. faecalis* for closed and open stored bottles.

The statistical analysis of *Ent. faecalis* showed no significant difference between the different concentrations of CO_2_ (Figure 7). For the open stored bottles, the median was 4 CFU/100mL.

## 4. Discussion

Among all contaminants of the water distribution system, such as inorganic and organic compounds, microbial contamination can lead to severe consequences for public health [61,62,63,64,65]. The intake of bacteria during the process of filling and capping bottles may lead to a reduced quality of bottled water, which is of interest to consumers as well as to its producers [29,66]. A study by Bedada et al. showed that molds were present in 15 of 44 examined bottled water brands. In one of the investigated samples, *S. aureus* and *Escherichia coli* were observed [42]. The Centers for Disease Control and Prevention (CDC) indicates: “Staph food poisoning is a gastrointestinal illness caused by eating foods contaminated with toxins produced by the bacterium *Staphylococcus aureus*”. The ability of *S. aureus* to poison food makes it necessary to avoid contamination [67]. Among different bacteria inhabiting acne vulgaris lesions, *S. aureus* was observed in a number of samples shown in a review on the microbiome of acne vulgaris in Indonesia [11]. Contamination of bottled water with *S. aureus* during the process of filling may happen from human skin during repairing or adjusting the machinery. The artificial contamination with *S. aureus* in our study, over a storage period of 30 days, showed a decrease in CFUs for all three levels of CO_2_ concentration (3.0 g/L, 5.5 g/L, and 7.0 g/L) within 18 days. After this period of time, no cultivable CFUs were detectable on Columbia Agar with 5% Sheep blood (BD^®^ Austria GmbH, Vienna, Austria). A study investigating the influence of pH on *S. aureus* growth indicated that pH values between 5.5 and 7.0 were suitable for *S. aureus* cultivation, similar to the results for *Staphylococcus epidermidis* [68], which stands in contrast to the findings by Iyer et al. [3]. In their study, *S. aureus* was more sensitive to the pH value. Our findings confirm that the survival of *S. aureus* may not depend on the provided pH value but on the carbonated mineral water on the media itself. Samples from the closed-stored bottles contained cultivable CFUs at all three concentrations for 16 to 18 days. In the open stored bottles with CO_2,_ bubbling out over time, cultivable CFUs of *S. aureus* were detectable for nearly one month. A recently published study showed that *S. aureus* (68%) and *Ent. faecalis* (68%) are present in household water storage tanks in Lebanon [69]. In 2005, Raj described the influence of storage temperature on bacterial growth in open stored bottled water. He concluded that the storage temperature has an enormous influence on bacterial counts [70]. Watanabe et al. examined the influence of the storage temperature of unfinished beverages (e.g., mineral water) on yeast, molds, and bacterial growth. The growth (< 3 log CFU) of *Ent. faecalis* and two other cocci in mineral water were described as not significant [71]. In our study, concentrations of 3.0 g/L CO_2_ and higher reduced the number of cultivable CFUs from approximately 40 on the first day to zero after the 13th day. Single CFUs were detected until the 13th day of storage in the closed stored bottles. A dependence of bacterial growth and pH value for *Ent. faecalis* in a highly alkaline environment was shown in a study by McHugh et al. [72]. A low pH environment may induce damage in the bacterial membrane, and/or nucleic acids and may lead to enzyme misfolding or denaturation [73]. Zhang et al. indicate that the molecular pathways of *Ent. faecalis* remain largely unknown [74]. Isolates of *Ent. faecalis* from healthy Chinese infants were found to survive at pH 5.0, in 7.0% NaCl, and in 3% bile salt for 20 h [75]. The exposure of *Ent. faecalis* in bottled carbonated mineral water with pH values from 5.4 to 6.4, over a period of many days, seems to reduce the ability to grow on Slanetz and Bartley Agar. The effect of pressurized CO_2_ on *Ent. faecalis* in different media was examined by Erkmen in 2000. The reduction in the pH value during the procedure of carbonation was described as well as the reduction in bacterial load at temperatures above 35 °C [76]. Storage temperatures above 35 °C combined with high CO_2_ pressure may reduce bacterial loads of *Ent. faecalis* faster than with the temperatures of 20 °C described in our study. The slower decrease in CFUs (*Ent. faecalis*) derived from samples from the open stored bottles can be described with the release of CO_2_ from the water and potentially raising pH values during this process. The sample design (CO_2_ concentrations, storage temperature, nutrient-poor water (total organic carbon <1.0 mg/L), etc.,) described in our study meets the conditions of production, distribution, disposal, and household storage of bottled carbonated mineral water. The findings of this study suggest that bottled carbonated mineral water with at least a concentration of 3.0 g/L CO_2_ and stored over a period of 30 days in glass bottles present no cultivable CFUs for *S. aureus* and *Ent. faecalis*. However, proper disinfection of all machinery and used glass bottles cannot be replaced, which ensures that all hygienic and technical standards are met. The consumer’s safety should be the most important aim despite the costs of additional treatments. Further studies including more pathogenic and facultative pathogenic microorganisms should be performed to investigate their behavior under the described conditions. According to the World Health Organization, microbial contamination of drinking water (tap water and bottled water) and irrigation water leads to risks to public health [77]. The addition of at least 3.0 g/L CO_2_ to bottled mineral water, a low-cost procedure, to assure a safe water supply should be considered in regions with problems concerning microbial contamination. The addition of CO_2_ should not be the only procedure executed to circumvent the problem of bacterial contamination in bottled water. Many questions regarding other pathogenic bacteria and the properties of fungal contamination stay unanswered. Future works should give attention to the behavior of Gram-negative bacteria and fungi under the described conditions.

## 5. Conclusions

During a period of 30 days, carbonated (3.0 g/L CO_2_, 5.5 g/L CO_2_, and 7.0 g/L CO_2_) bottled mineral water samples, which were artificially contaminated with either *S. aureus* or *Ent. faecalis*, two Gram-positive bacteria, were examined for bacterial growth. A decrease in the number of CFUs was observed for all samples during the investigation period. The pH value dependence of the examined bacterial strains can describe the observed decrease in CFUs over several days of storage. Further investigations for other microorganisms that can act as contaminants should be made in the future, to complete the whole picture of bottled water contaminant behaviors.

## Figures and Tables

**Table 1:** Contaminated mineral water bottles with different concentrations of CO_2_.

Number of Bottles (250 mL)	Concentration of CO_2_ (g/L)	Bacterial Strain
20	3.0	*Ent. faecalis* ATCC 6057
40	5.5	*Ent. faecalis* ATCC 6057
20	7.0	*Ent. faecalis* ATCC 6057
20	3.0	*S. aureus* ATCC 25923
40	5.5	*S. aureus* ATCC 25923
20	7.0	*S. aureus* ATCC 25923

## Data Availability

Not applicable.

## Supplementary Materials

The following supporting information can be downloaded at: https://www.mdpi.com/article/10.3390/biology12030432/s1, Table S1: Bacterial counts.
